# Debate: the per rectal/digital rectal examination exam in the emergency department, still best practice?

**DOI:** 10.1186/s12245-018-0165-z

**Published:** 2018-03-27

**Authors:** John Quinn, Tomas Zeleny, Venu Rajaratnam, Dan-Lucian Ghiurluc, Vladimir Bencko

**Affiliations:** 1 Prague Center for Global Health, Institute of Hygiene and Epidemiology, First Faculty of Medicine, Charles University in Prague, Prague, Czech Republic; 2 Institute of Hygiene and Epidemiology, First Faculty of Medicine, Charles University in Prague, Prague, Czech Republic; 3 SHO General Surgery, Obstetrics/Gynaecology, Morecambe Bay Hospitals Trust, Kendal, UK; 4 Research Fellow, Prague Center for Global Health, Prague, Czech Republic; 5 Northwick Park Hospital, London, UK

## Abstract

**Background:**

Emergency medicine practice in the UK and Ireland offers a junior and middle grade doctor great learning opportunities that force engagement with multiple specialties, life-saving procedures, exposure to a myriad of patient presentations, and opportunities for best practices in medicine.

**Main body:**

The emergency department (ED) can be a hectic and dynamic environment; communication from the ED to specialists is essential to ensure best clinical outcomes for patients. The “per rectal” (PR) or “digital rectal exam” (DRE) can be a very difficult diagnostic test for even the most skilled operator to discern pathological versus normal; we propose this is especially the case in the emergency department patient population. Some specialists require this exam performed by an unskilled junior doctor with varying results prior to reviewing a referred and sick patient. The PR/DRE benefits may be limited in the ED setting for some pathologies, and the result of the exam may have limited impact in the overall treatment plan in the ED.

**Conclusion:**

This short paper reviews the indications, benefits, shortfalls, and limitations of the PR/DRE in the emergency department setting and offers novel alternatives to maximize best practice, ensure best clinical outcomes for patients, and, to first, do no harm.

## Introduction

The digital rectal exam (i.e., “PR” or “DRE”) is not a clinical skill that junior and many middle grade doctors in the ED enjoy the same way as inserting a central line with ultrasound guidance, intubation, or reduction of a Colles’ fracture liberating instant clinical gratification. The PR or DRE is not at all comfortable or welcomed by an overwhelming majority of patients. However, not all medicine is comfortable and there are risks and benefits to all clinical interventions and required diagnostics, and the PR/DRE exam may provide pellets of diagnostic information that can change or impact patient care and management. The PR/DRE exam falls into a category of diagnostics that can be objective and subjective in nature, but its decision-making value alone is extremely limited.

This is not to take away the absolute requirement of all physicians to be good diagnosticians and place hands onto the patient to aid the patient-physician interaction and therapeutic relationship. Technology alone will not be the panacea for medicine and clinical practice rendering the physical exam obsolete. Good clinical practice requires sound decision-making and doing the right test at the right time to guide patient care. The ED may not be the ideal setting for blind PR/DRE based on the clinical picture before specialist review. The ethos of the clinical sage Dr. William Oslers’ words “One finger in the throat and one in the rectum makes a good diagnostician,” may not have to be followed to the letter of the law in the ED setting with its very diverse patient population [1].

## The review

The PR/DRE is one tool in the diagnostic arsenal along with percussion, palpation auscultation, etc. In the hospital patient population, many treating physicians are so adamant about the exam, some lament, “It should be considered in all patients admitted to hospital who are over the age of 40, unless the examiner has no fingers, the patient no anus, or acute illness such as myocardial infarction presents a temporary contraindication” [2]. Indeed, current best practices require the PR/DRE exam to be performed [3, 4]. The purpose of this review paper is to challenge some of these branches in the diagnostics and treatment algorithm of the undifferentiated patient and to consider the clinical risks and benefits but also alternatives to the exam.

In conducting the diagnostic procedure, performed in the ED or on a specialty ward, as with all diagnostics, patient consent must be obtained. Further, privacy must be ensured; a chaperone is always indicated in the emergency department setting and a simple explanation to the patient about the exam, why the exam is being performed and discussion of an alternative if the patient refuses the exam. The patient is placed on their side with the knees pulled towards their chest into the left lateral position. The lubricated and gloved hand of the examiner begins the inspection of the anus and perianal area by separating the buttocks. Observation for thrombosed external hemorrhoids, skin tags, rectal prolapse, fistula-in-ano, condylomata acuminata, carcinoma of the anus, and pruritus ani are examined and noted—all without penetrating finger into anus. Of course, gross blood at the anus, acute and obvious trauma, or incontinence are also observed and ambiance appreciated at this stage.

To perform the exam intrarectally, the tip of the gloved finger is lubricated and placed over the anus, the patient can be encouraged to breathe out. The examiner introduces the finger and palpates for anal fissures, thrombosed external or internal hemorrhoids, an ischiorectal abscess, active proctitis, or anal ulceration [4]. The finger is then rotated so that the left lateral wall, posterior wall, and right lateral wall of the rectum can be palpated in turn; then, the finger is advanced as high as possible into the rectum and slowly withdrawn along the rectal wall. A soft lesion, such as a small rectal carcinoma or polyp, is more likely to be felt with this technique on exit [2].

The patient can then be asked to squeeze the examiner’s finger when still in situ with the anal muscles as a further test of anal tone [4]. This subjective phase of the exam does not account for the depth or width of the examiner’s fingers. It should be noted that among medical students and practicing consultants, no data has been collected or reported on average or maximum/minimum finger girth or length to date.

However, this exam can tell if sphincter tone is present based on the sensation of the examiner alone, if the rectal wall has been breached, if spicules of bone are palpable, if the prostate in a normal position, and subjectively if there is gross blood on the examiner’s finger [5]. Specifically, “the rectal examination may elicit reduced tone from spinal cord injury, bleeding from lower gastrointestinal trauma, bony penetration in pelvic fracture and a high-riding prostate associated with pelvic fracture, an indicator of potential urethral injury” [5]. The presence of rectal tone or variations in rectal tone are poorly reported. In sum, a rectal examination is performed to look for blood, lack of tone/sensation, or high-riding prostate which indicate bowel injury, spinal cord injury, or urethral injury, respectively—the rectal examination can be an important part of the examination, but stool must be tested for blood.

## The blood

The core issue with hematochezia and confirmation of same is where in the fecal flow the test sample is taken. Blood can be elicited in the patient complaining of or not complaining of hematochezia inside the rectum or in feces produced in the ED. The index of suspicion for the patient presenting with a marked self-reported history of blood or black tarry stools to the ED should be high for hematochezia. The level of suspicion and disbelief by some physicians is worrisome and not duplicated or seen in patients presenting with a history of chest pain, abdominal pain, and other subjective presentations to the ED as it is for a history of hematochezia. Believing, or at least taking seriously such reports of presenting patient complaints, is the core tenets in the patient-physician relationship.

Localized bleeding in or near the anal canal is commonly caused by anal fissures or hemorrhoids. If the patient is well, they can be reassured in the ED, but patients should have follow-up to exclude more serious causes. Inflammatory bowel disease, diverticulitis, and lower gastrointestinal (GI) carcinoma should be considered in patients who are unwell [6]. The need to analyze stool for blood, in small microscopic amounts, is obvious and easily done with a simple, cheap, and non-invasive test: the fecal occult blood test.

## The test

### Stool for occult blood (stool for OB, fecal occult blood test [FOBT], fecal immunochemical test [FIT], DNA stool sample)

This test can be used for colorectal cancer screening of asymptomatic individuals, occult blood from other causes such as ulcers, hemorrhoids, diverticulosis, and others [7]. Normally only minimal quantities (2 to 2.5 mL) of blood are passed into the gastrointestinal (GI) tract; this bleeding is not significant enough to cause a positive result in the stool for occult blood (OB) which can detect as little as 5 mL of blood is lost per day [7]. Guaiac is the most commonly performed chemical assay found in the literature. The peroxidase-like activity of hemoglobin catalyzes the reaction of peroxide and a chromogen called orthotolidine to form a blue-stained oxidized orthotolidine [8].

OB can also be detected by immunochemical methods that detect the human globin portion of hemoglobin using monoclonal antibodies. These tests are called fecal immunochemical test or immunochemical fecal occult blood test (iFOBT) [7]. At many EDs throughout Ireland and the UK, bedside and lab test to confirm blood in the stool are not readily available. This simple test may support clinical decision-making when used with sound clinical judgment. In sum, without objective data from a FOBT, the PR/DRE can be performed with no definitive diagnostic value.

## The standards

There are clinically different diagnostic requirements for the acute trauma and medical patients and the subacute or clinically well patient presenting that may require a PR/DRE. For example, we have already seen above that some medical requirements may lean towards the side that all medical patients in hospital undergo the exam. For trauma, the eight edition of advanced trauma life support (ATLS) recommends that “DRE be performed selectively before inserting an indwelling urinary catheter” [9]. However, in trauma patients, the role of the PR/DRE rarely changes the management. PR/DRE was felt to change management in only in 1.2% of cases in Porter and Ursic’s prospective observational study (2001) and only 4% in Esposito et al.’s prospective study (2005) looking at the clinical need and change in management from the outcome of the DRE [10, 11]. Esposito et al. [10] found that none of 512 patients would have had a significant injury missed had the PR/DRE been omitted.^1^ The PR/DRE has a limited role in the evaluation of the general trauma patient. Said plainly, when it is done and positive, it may influence or change management; however, even if it was not done, the pathology found would be found though alternate less invasive mechanisms such as blood results and imaging, rendering the DRE/DR exam unneeded in many patient groups.

The sensitivities of the exam can be high, meaning positive in disease, but its low likelihood of finding pathology make it a poor screening exam. Currently, its utility as the screening exam ATLS intended it to be is not supported with current data.

## The patient experience

No patient wants a stranger to digitally exam the inside of their rectum. Patients routinely oblige for training and diagnostic purposes with the idea that benefits outweigh the “discomfort.” There are instances of patients who have had relatives die as a result of prostate cancer or surviving patients, offer their rectums and insides to science for medical students to practice. The benefit being that if you can practice on a normal prostate or PR/DREE exam, it will offer an epiphany to the abnormal when it is encountered by junior doctors, or that when an anal fissure is felt, it would not be missed.

Other patient experience is far more controversial and led to assault and battery in the alert and aware trauma patient [12]. Is there that much clinical value in the PR/DRE that wrestling and eventually intubating a patient to perform a PR/DRE is necessary? Have clinicians forget alternatives to diagnostics that have limited value in the ED/trauma setting? It may be that many clinicians and local protocols need to rethink the process and requirements.

## The debate

Why perform this finger-wave test that may cause trauma to the patient, is minimally valuable, and may not change patient care plans and management? The insanity is that many referral receiving physicians will not accept a patient without such a test being performed. Clinically, it is difficult to see where the subjective findings of PR/DRE dictate further or change treatment.

For example, the patient presenting to the ED with a 6-month history of black tarry stools and a Hb = 8.2 g/dL (new for this patient from trend blood values that have been at 13 g/dL as recent as 6 months prior, before the black tarry stools began) with mild abdominal discomfort, no history of trauma and history of a 30 pack year for smoking filterless cigarettes, alcohol misuse, and family history of colorectal cancer, who has three servings of home-cooked smoked sausage a day since birth from Moravia. For this patient, which is more diagnostic, the PR/DRE exam or the objective and new findings of Hb = 8.2 g/dL requiring further investigation, possibly as inpatient in the clinically unwell patient? Where does the PR/DRE exam help in the management decisions for this patient? An external exam for gross hemorrhoids, acute, bright red blood, and active bleeding can certainly rule out the obvious, and there was no history for bright red blood. Simply put, any patient with an Hb = 8.2 g/dL requires further evaluation despite the results and subjective findings of a PR/DRE. Conversely, if upper gastrointestinal hemorrhage is suspected, some value of a PR/DRE can be gleaned and appropriate management given if results are forthcoming.

In the same patient presentation that is stable and clinically well with Hb = 15 g/dL, how would the PR/DRE exam help? Would a subjective bloody finger, internal hemorrhoids, or other finding change the need for outpatient management of this otherwise stable patient? If the occult blood test were performed in the clinically well patient and was positive, would outpatient management still be warranted? So, where was the PR/DRE exam helpful and why did we do it? What will be definitive for both patients in relation to any abnormality, a thick finger inserted 4 cm into the rectum or a camera to the ascending colon with digital or further contrast imaging, reproducible and objective? Most endoscopies involve anesthesia of some kind and are performed in the non-ED environment.

## The risk

It hurts; no one likes it; in prison, it is a form of rape; patients get angry during the exam when confused or in high stress, and it can lead to medical malpractice issues. There is a possible infection risk from contamination of local wounds; the risk of transmission of infection to the clinician can occur; and the risk of injury—potential for worsening of the patient’s injuries with unstable pelvic fracture and rectal defects—and also risk of injury to the clinician with any body cavity exploration from foreign bodies, bone fragments, etc. must be considered. And it should be noted that there is no evidence in the current literature that addresses infection risk of the routine or emergency rectal exam in the emergency department setting for patient or clinician. Finally, the possibility of false positive and false negative DRE findings, along with requirements to repeat the exam by other specialities, limits its value and increases risk.

## The alternative

As clinicians, it is our responsibility and duty to offer patient alternatives to treatment, diagnostics, and education about the risks and benefits of both. The paternalistic Dr. Evil that snaps a gloved finger asking the patient to bend over is no longer tolerated or best practice. An alternative may be found within the Guaiak Sleeve. No more than half a finger in size, the Guaiak Sleeve has practitioners simply place the sleeve on the examiner’s finger, perform the exam minimally, and quickly get results on whether stool is occult blood positive or negative [13]. This alternative may offer a less invasive modality to conclude on bleeding and minimize patient discomfort.

## The evidence

The DRE has been shown to have poor sensitivity as a screening exam, yielding high false-negatives in diagnosing index injuries and urethral disruptions, and based on physician survey, DREs only add pertinent information in an estimated 5% of cases and change management in 4% of cases [14]. Some evidence of the utility is controversial.

Another purpose of the DRE is to measure anal sphincter tone; however, anal sphincter tone correlates poorly with the “criterion standard” of manometry, yielding poor sensitivity and positive predictive value. When examining the diagnosis of acute appendicitis in both children and adults, studies have concluded that DREs give no significant direction in diagnosis and can be most likely omitted in the evaluation of acute undifferentiated abdominal pain and acute appendicitis [14].

In a simple review article searching the literature for utility of the DRE in evaluating acute, undifferentiated abdominal pain, suspected appendicitis, fecal occult blood, and anal sphincter tone, Kessler and Bauer found that the PR/DRE not only confound but also add no additional information to a physician’s diagnosis or management in the emergency department [15]. It should be noted that the use of the DRE in colorectal neoplasm and benign prostatic hyperplasia screenings was beyond the scope of the article and were not included in the literature review methodology. The paper also reported that the sensitivity, specificity, positive predictive value, negative predictive value, and odds ratio for the PR/DRE were too low to reliably diagnose acute appendicitis in children and adults [15].

Conversely, Orkin and colleagues also identified that assessment of anal sphincter tone is a critical part of anorectal examination, “yet no standardized, quantifiable method for describing anal sphincter tone on digital rectal examination exists,” which led to the development of a novel scoring system for sphincter tone using a scale of 0 to 5 [16]. They hypothesized that the digital rectal examination scoring system (DRESS) score would correlate with anorectal manometry pressures. After reviewing roughly 300 patients in the controlled hospital setting, they found that their proposed “DRESS score” correlated very well with manometry pressures for resting and squeeze pressures [16].

However, in the emergency department population, and in much less controlled settings, they also concluded the limitations of their findings that further research and validation are required. This is especially so in order to support adoption of such a system as part of any standard anorectal examination, although further standardization and validation in the emergency department population would be a great addition into the efficacy and use of this diagnostic tool for the acute patient.

## The discussion

In summary, emergency physicians should implement selective, rather than discretionary, use of the DRE. As emergency department clinicians, chief complaints, physical exam, and clinical judgment dictate our clinical impression and treatment plan. We need to think, where the PR/DRE fits, if at all, into the treatment plan of action and is it needed. There is utility in the PR/DRE exam in specific patient histories and presenting complaints. These patients should still have the PR/DRE considered to aid in the diagnostic and clinical care and treatment plan.

## The conclusion

In sum, the reflex of the PR/DRE exam in the undifferentiated abdominal pain, rectal bleed, and trauma for patients presenting to the ED may need to be reviewed. The utility in certain presentations is very limited and, despite many specialities unwillingness to accept to review or admit a patient based on this invasive test being performed, may also need to be reviewed to ensure best practice and best outcomes. Patients present to the ED for a multitude of reasons, in providing them best clinical practice, we must recall the words that christened all of our medical licenses, “first do no harm,” when considering any diagnostic procedure.

## Abbreviations

DRE: Digital rectal exam
ED: Emergency department
PR: Per rectal

## Footnotes

1 Please see more detailed reports at: http://lifeinthefastlane.com/trauma-tribulation-012/
